# Group Music Activities and Academic Burnout Among Non-Music-Major College Students with Recent Group Music Participation: A Cross-Sectional SEM–fsQCA Study

**DOI:** 10.3390/bs16101887

**Published:** 2026-10-13

**Authors:** Lingran Liu, Haohao Yang, Sen Chen

**Affiliations:** 1 School of Art, Southeast University, Nanjing 211102, China; 2 School of Music, Shaanxi Normal University, Xi’an 710119, China; 3 Institute of Sports Science, Sichuan University, Chengdu 610065, China; rockychensen@126.com

**Keywords:** academic burnout, group music activities, social connectedness, collective effervescence, serial mediation, fsQCA, cross-sectional study, non-music-major college students

## Abstract

Academic burnout among college students is an important concern in educational psychology and student well-being. This cross-sectional study examined the associations among group music activity participation, social connectedness, collective effervescence, and academic burnout among non-music-major college students with recent group music participation. A questionnaire survey was conducted among 1027 college students in China who had participated in at least one group music activity during the previous semester. Covariance-based structural equation modeling (CB-SEM) was used to examine whether group music activity participation was associated with academic burnout directly and indirectly through social connectedness and collective effervescence, including a proposed sequence from social connectedness to collective effervescence. Fuzzy-set qualitative comparative analysis (fsQCA) was further used to examine which combinations of group music activity participation, social connectedness, and collective effervescence were associated with low academic burnout. The SEM results showed that higher group music activity participation was significantly associated with lower academic burnout. Social connectedness and collective effervescence were involved in significant individual indirect associations, and the serial indirect association involving social connectedness followed by collective effervescence was also statistically supported. The fsQCA results showed that no single condition constituted a necessary condition for low academic burnout. The joint presence of high group music activity participation, high social connectedness, and high collective effervescence formed a sufficient configuration associated with low academic burnout (consistency = 0.896, coverage = 0.647) under the present sample and analytical conditions. Taken together, the findings suggest that group music participation, relational connectedness, and shared emotional experience are meaningfully associated with differences in academic burnout among non-music-major college students. These findings provide a complementary variable-centered and configurational perspective on the psychosocial correlates of group music participation and may inform future longitudinal research and campus-based student well-being initiatives.

## 1. Introduction

Academic burnout has become an important topic in research on college students’ psychological adjustment (El Barusi & Kurniawati, 2024). At the university level, students face multiple academic and developmental demands, including coursework, performance evaluation, further study, employment, and social comparison (Barbayannis et al., 2022; W. Gao et al., 2020; Kumaraswamy, 2013; Park et al., 2023). Under these sustained demands, some students may experience substantial exhaustion (Liu et al., 2023). Academic burnout is also associated with lower subjective well-being and poorer mental health (X. Cheng & Lin, 2023; Mudło-Głagolska & Larionow, 2025). Accordingly, identifying factors associated with academic burnout and clarifying the psychosocial processes related to its occurrence and variation remain important tasks in educational psychology.

Previous studies have examined academic burnout from multiple perspectives, including perceived stress, personality traits, coping styles, psychological capital, and social support (Biaigo et al., 2025; Cuevas-Caravaca et al., 2024; X. Gao, 2023; Vîrgă et al., 2022; Ye et al., 2021). These studies provide an important basis for understanding the individual and interpersonal correlates of burnout. However, existing explanatory frameworks have often emphasized individual-level factors, with comparatively less attention to shared experiences embedded in the social contexts of students’ everyday lives. College students’ academic adjustment is related not only to intra-individual characteristics but also to their interpersonal interactions and campus experiences (López Turley & Wodtke, 2010; Yin & Shi, 2022). A sense of connection with others and the emotional experiences arising from shared activities may therefore be relevant to students’ psychological adjustment (Glass et al., 2014). From this perspective, collective participation may represent an important social context in which psychosocial resources related to academic burnout can be examined.

Among the many forms of collective participation available on university campuses, group music activities provide a distinctive context for examining these processes. Social connectedness, emotional sharing, and group belonging may also arise in sports, drama, dance, volunteering, student clubs, and other forms of collective activity. Group music activities are therefore not assumed to be unique in their capacity to support these psychosocial experiences. However, they may provide a particularly salient context because they commonly involve rhythmic synchronization, shared sound production, vocal or instrumental coordination, bodily movement, and coordinated emotional expression. Unlike the relatively private experience of listening to music alone, group music activities emphasize shared participation, mutual responsiveness, and coordinated action. These characteristics may make relational and emotional aspects of collective participation especially visible within musical settings.

Despite growing interest in the psychosocial value of group music participation, relatively little research has focused specifically on its association with academic burnout among college students. Further research is therefore needed to clarify how group music participation is related to students’ academic adjustment within everyday campus life. In addition, lower academic burnout may not be associated with a single factor or a single linear pathway. Different combinations of behavioral, relational, and emotional conditions may correspond to lower burnout, suggesting that both variable-centered and configurational perspectives may be useful for examining these relationships.

Against this background, the present study examines the associations among group music activity participation, social connectedness, collective effervescence, and academic burnout among 1027 Chinese college students. Structural equation modeling (SEM) is used to evaluate the direct and indirect associations specified in the proposed model, including the serial indirect association involving social connectedness and collective effervescence. Fuzzy-set qualitative comparative analysis (fsQCA) is used to examine configurations of conditions associated with low academic burnout. By combining these two approaches, the study aims to provide complementary variable-centered and configurational perspectives on the psychosocial correlates of group music participation. The findings may contribute to a more contextually grounded understanding of academic burnout and provide a basis for future longitudinal research and campus-based student well-being initiatives.

## 2. Literature Review

### 2.1. Existing Evidence on Group Music Activity Participation and Academic Burnout

Group music activity participation refers to individuals’ involvement in choral, ensemble, and other forms of shared musical practice within a group context. Such activities typically require participants to perceive others’ actions, coordinate timing and rhythm, and continuously adjust their own responses during the shared musical process. Group music activities can therefore be understood not only as artistic practices but also as forms of collective participation that provide opportunities for interaction, coordination, and relational experience (Schiavio et al., 2022).

One distinctive characteristic of group music making is the prominence of interpersonal coordination. Participants often need to coordinate rhythm, tempo, sound intensity, breathing, bodily movement, and expressive timing. Keller (2023) emphasized that musical group interaction depends on synchronization, synergy, and coordinated collective action that cannot be reduced to the simple addition of individual behaviors. Similarly, Demos and Palmer (2023) described musical group synchrony as involving both social and nonlinear dynamics, highlighting the continuous coordination that develops as participants adjust to one another during shared musical activity. These characteristics make group music activities a particularly salient context for examining interpersonal interaction and collective coordination.

The social relevance of group music participation has also been supported by previous research. Schellenberg et al. (2015) found that group music training was associated with greater empathy and prosocial behavior among children, suggesting that the psychosocial relevance of group music participation may extend beyond musical skill itself. Bartleet and Pairon (2021) similarly emphasized the positive social significance of participatory music practices in educational, community, and social settings. Taken together, these studies suggest that group music activity participation may provide a context in which connection, collaboration, and social engagement can develop through shared musical practice. This does not imply that such relational processes are unique to music; rather, music provides a distinctive embodied and affective setting in which coordination and shared participation are especially salient.

Academic burnout refers to a negative psychological state associated with prolonged academic demands and sustained learning pressure (Maslach et al., 2001; Schaufeli et al., 2002). Existing research commonly conceptualizes academic burnout as involving emotional and motivational difficulties that emerge under sustained academic strain. Ghods et al. (2023) described academic burnout within the three-dimensional framework of exhaustion, cynicism, and reduced efficacy. Berjot et al. (2022) further noted that burnout in students may be reflected in exhaustion during the learning process, psychological detachment from academic tasks and relationships, and a reduced sense of academic competence.

Research on academic burnout has increasingly emphasized the importance of both academic demands and the psychosocial resources available to students. Earlier studies identified academic stress as an important correlate of academic burnout (Kim et al., 2015), while subsequent research has shown that interpersonal and psychological resources may also be relevant. J. Cheng et al. (2020), for example, found that resilience and social support buffered the association between academic burnout and depression among Chinese medical students. Jung et al. (2015) similarly reported that academic self-efficacy partially mediated the association between academic stress and academic burnout among Chinese adolescents. These findings suggest that academic burnout is related not only to the level of academic demands but also to the psychosocial resources available to individuals when coping with those demands.

Within this broader framework, group music participation may be relevant because of its interactive, collaborative, and socially coordinated characteristics. Research among Chinese college students has shown that participation in group musical activities is associated with musical self-expression, empathy, and prosocial behavior, suggesting potential links with positive social adjustment and interpersonal connectedness (Qiao, 2026). Acquah (2016) also reported that choral participation among college music students was associated with better emotional, psychological, and social well-being. These findings provide a basis for considering whether the relational and shared experiential qualities of group music participation may also be associated with academic burnout.

Accordingly, the present study proposes that higher levels of group music activity participation will be associated with lower levels of academic burnout among college students. This hypothesis concerns an association rather than a causal effect, consistent with the cross-sectional design of the present study.

**H1.**  *Group music activity participation is negatively associated with academic burnout.*

### 2.2. The Mediating Role of Social Connectedness

Social connectedness generally refers to an individual’s perceived sense of connection, belonging, and psychological proximity to other people, groups, or broader social communities (Hein et al., 2024; Thomas & Griffin, 2023). Plesko et al. (2021) similarly emphasized belonging and connection to others or to a community as central features of social connectedness. This conceptualization highlights the subjective and relational nature of the construct.

Among student populations, social connectedness has been identified as an important psychosocial resource associated with psychological adjustment. Mickley Steinmetz et al. (2024) found that higher levels of social connectedness were associated with lower examination stress among college students. L. Tang et al. (2025) likewise reported that social connectedness was associated with better psychological well-being among international students, partly through lower loneliness and perceived stress. Using longitudinal data, Sun et al. (2021) further showed that social connectedness buffered the association between discriminatory experiences and symptoms of anxiety and depression among Chinese international students. Taken together, these studies suggest that social connectedness may be relevant to how students experience and respond to stressful social and academic contexts.

Social connectedness may also help explain how experiences within social environments are associated with broader adjustment outcomes. Avcı (2023) found that social connectedness partially mediated the association between belongingness and life satisfaction among college students, suggesting that perceived connection and acceptance may be one pathway linking social experience with psychological adjustment. Yıldırım et al. (2021) similarly reported that social connectedness was involved in the association between stress and burnout among healthcare workers. Although these studies were conducted in different populations and contexts, they support the broader view that social connectedness may function as an important psychosocial correlate of adjustment under stressful conditions.

This perspective is also relevant to group music participation. Group music activities are inherently collaborative and require repeated interaction, coordination, and mutual responsiveness. Weinstein et al. (2016) showed that group singing was associated with social bonding, suggesting that shared musical participation may provide opportunities for participants to experience stronger interpersonal connection and group belonging. Within the context of the present study, these relational experiences may be particularly relevant because academic burnout is associated not only with academic demands but also with the psychosocial resources available to students when coping with those demands.

Accordingly, group music activity participation may be indirectly associated with lower academic burnout through its positive association with social connectedness. In this proposed indirect association, participation in group music activities is expected to correspond to stronger social connectedness, which in turn is expected to be associated with lower academic burnout. This interpretation concerns an indirect association rather than an established causal mechanism, consistent with the cross-sectional design of the present study.

**H2.**  *Social connectedness is expected to mediate the association between group music activity participation and academic burnout.*

### 2.3. The Mediating Role of Collective Effervescence

Collective effervescence refers to a state of heightened shared emotional activation and group unity that may arise during rituals, celebrations, or other forms of collective gathering (Rimé & Páez, 2023). Pizarro et al. (2022) described collective effervescence as involving emotional synchronization, joint attention, and a strong sense of shared emotional activation and unity. In this sense, individuals experience emotion not only at the personal level but also as part of a shared group experience.

The emergence of collective effervescence is closely related to situational conditions that facilitate co-presence and shared attention. Liebst (2019) found that collective effervescence is associated with crowd co-presence and shared participation, suggesting that collective emotional experiences may intensify when individuals are physically and socially engaged in the same situation. In live music contexts, Swarbrick and Vuoskoski (2023) reported that co-presence may strengthen audience connectedness and collective experience through physical synchronization and emotional resonance. Rickard et al. (2025) similarly noted that live music events are associated with shared experience, group empowerment, and a sustained sense of community. Together, these findings suggest that collective effervescence can be understood as a social-emotional experience associated with coordinated participation and shared emotional engagement.

This interpretation is consistent with Durkheim’s conceptualization of collective effervescence. Durkheim proposed that participation in rituals or collective gatherings may be accompanied by heightened emotion and a sense of group unity that exceeds ordinary individual experience (Durkheim, 2016). Subsequent research has likewise emphasized synchronization and co-participation as important conditions associated with shared collective experience (Mogan et al., 2017). Within group music activities, these processes may be particularly salient because participants coordinate rhythm, sound production, bodily movement, attention, and emotional expression within the same shared activity. These characteristics do not imply that collective effervescence is unique to music, but they make musical participation a useful context in which such shared emotional processes can be examined.

Research has further suggested that collective effervescence is associated with positive psychosocial outcomes. Otsu et al. (2021) and Mentel (2022) emphasized the relevance of co-presence, attention, rhythm, and synchronization to collective emotional experience. Wlodarczyk et al. (2020) and Naidu et al. (2024) likewise linked collective effervescence with outcomes such as well-being and social integration. These findings suggest that stronger collective emotional experiences may be associated with more positive psychological states, although the direction and temporal ordering of these relationships cannot be established from the present cross-sectional design.

Within the context of academic burnout, collective effervescence may therefore represent one emotional pathway through which group music participation is associated with students’ academic adjustment. Group music activities provide opportunities for shared attention, coordinated action, and emotional synchronization, while collective effervescence reflects the subjective experience of heightened shared emotion and group unity arising in such contexts. Accordingly, stronger collective effervescence may be associated with lower levels of emotional exhaustion and psychological withdrawal related to academic burnout.

Based on this reasoning, the present study proposes the following hypothesis:

**H3.**  *Collective effervescence is expected to mediate the association between group music activity participation and academic burnout.*

### 2.4. Serial Mediation of Social Connectedness and Collective Effervescence

Interaction ritual chain theory provides a useful framework for understanding how shared participation may be associated with relational and emotional experiences in collective settings. Collins (2014) proposed that successful interaction rituals are characterized by conditions such as co-presence, joint attention, emotional sharing, and boundaries distinguishing participants from outsiders. Under these conditions, collective interaction may be associated with outcomes such as emotional energy, group solidarity, symbolic meaning, and a sense of belonging. Related research has applied this framework to collective events. Xie and Li (2023), for example, found that interaction ritual conditions were associated with perceived emotional synchronization among festival participants, while Hill et al. (2022) showed that interaction ritual processes in live events can contribute to social attachment and shared group experiences.

This theoretical perspective is also relevant to group music activity participation, although the underlying processes are not unique to music. Like other collective practices, group music activities involve co-presence, joint attention, interaction, and emotional sharing. Their distinctive feature lies in the way these processes are organized through rhythm, shared sound production, vocal or instrumental coordination, and bodily synchronization. These characteristics may make group music activities a particularly salient context in which relational connection and shared emotional experience occur together (Warran, 2025; Van der Merwe & Morelli, 2022).

In accessible terms, the serial mediation model examined in this study asks whether group music activity participation is associated with academic burnout partly through two linked psychosocial experiences. Specifically, the proposed model examines whether greater participation in group music activities is associated first with stronger social connectedness, whether stronger social connectedness is in turn associated with greater collective effervescence, and whether these experiences are subsequently associated with lower academic burnout. Thus, rather than treating social connectedness and collective effervescence only as two separate mediators, the model also examines whether they form a theoretically ordered indirect association.

The proposed ordering from social connectedness to collective effervescence is theoretically grounded in the distinction between relational integration and shared emotional activation. Social connectedness reflects an individual’s perceived sense of belonging, acceptance, and psychological connection with others. Within group music activities, repeated coordination and mutual responsiveness may be associated with stronger relational ties and a greater sense of inclusion. Once participants experience stronger relational connection, they may be more likely to engage in shared attention and emotional synchronization during collective participation. In this sense, social connectedness may provide a relational context in which collective effervescence, characterized by heightened shared emotional activation and group unity, is more likely to be experienced.

This interpretation is consistent with previous research indicating that collective participation, shared attention, and emotional synchronization are closely related to social integration and group identity. Perceived emotional synchronization in collective settings is associated with social integration and positive psychological outcomes (Páez et al., 2015; Zabala et al., 2023). Research on live music experiences has likewise suggested that shared musical participation can foster social connectedness and contribute to psychological and social well-being beyond the immediate event (Packer & Ballantyne, 2011). Together, these studies provide theoretical support for considering relational and emotional experiences within the same indirect model.

**Figure 1:**
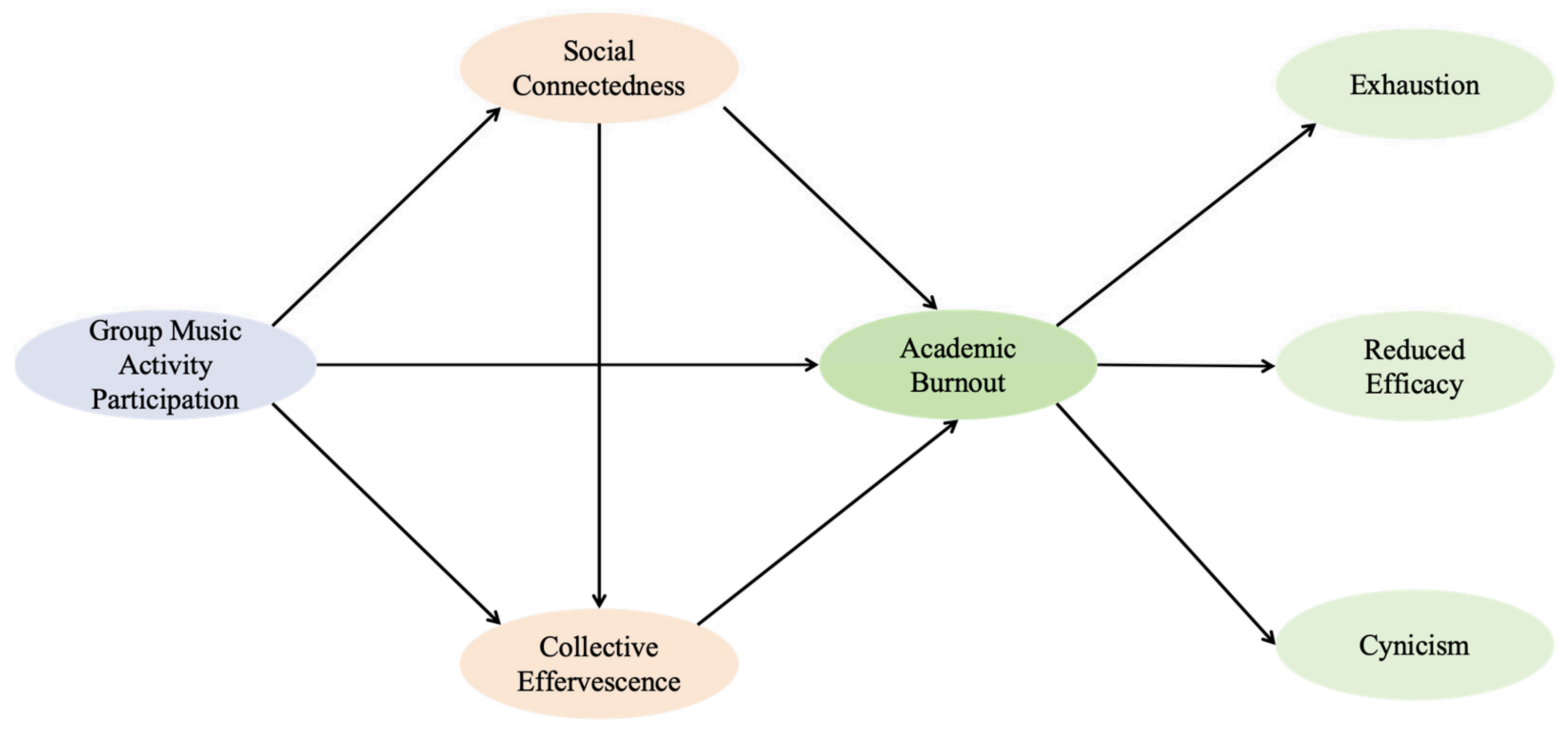
Proposed modeling diagram.

However, the proposed sequence should not be interpreted as an established temporal or causal mechanism. The present study uses cross-sectional data, and all core variables were measured at the same time. Therefore, the analysis cannot demonstrate that social connectedness temporally precedes collective effervescence. It is also theoretically plausible that intense shared emotional experiences may themselves strengthen participants’ subsequent sense of social connectedness. The ordering tested in this study should therefore be understood as a theoretically specified model derived from interaction ritual chain theory rather than as proof of a unidirectional psychological process. Alternative theoretically plausible specifications may also be statistically equivalent or yield comparable model fit; therefore, comparisons of model fit based on the present cross-sectional data cannot establish the temporal direction between social connectedness and collective effervescence. Future longitudinal or experimental research is needed to examine the temporal relationship between these two constructs more directly. Based on this reasoning, the following hypothesis is proposed (Figure 1):

**H4.**  *Social connectedness and collective effervescence are expected to serially mediate the association between group music activity participation and academic burnout, such that group music activity participation is positively associated with social connectedness, social connectedness is positively associated with collective effervescence, and collective effervescence is negatively associated with academic burnout.*

## 3. Materials and Methods

### 3.1. Respondents and Questionnaire Distribution

This study focused on full-time university students in China and employed purposive, site-based recruitment to conduct an offline questionnaire survey. Recruitment was conducted across six universities located in Jiangsu, Shaanxi, Sichuan, Beijing, and Guangdong, China: Southeast University, Nanjing Normal University, Shaanxi Normal University, Sichuan University, Beijing Normal University, and Shenzhen University. To protect participant privacy, individual-level identifiers linking respondents to specific universities or activity groups were removed during data cleaning and anonymization; therefore, only institutional-level information was retained.

The following eligibility criteria were applied. First, respondents were required to be full-time university students, including undergraduate and graduate students. Second, their major could not be in a field involving professional music training, such as musicology or music performance. Third, they were required to have participated in at least one group music activity on campus, such as choir or band rehearsals, campus singing competitions, or music club activities, during the previous semester. Fourth, they were required to be able to understand and complete the questionnaire independently.

Participants were recruited primarily from campus settings associated with group music activities, including non-professional music club events, choir rehearsals, campus competitions, and rehearsals for cultural performances. Before questionnaire administration, the researchers contacted relevant faculty members, advisors, and club leaders and, after obtaining their support, distributed the questionnaires on site. All questionnaires were administered in person using standardized instructions explaining the purpose of the study, the anonymity of responses, and the voluntary nature of participation. Screening questions were included at the beginning of the questionnaire to confirm that respondents were non-music majors and had participated in at least one group music activity during the previous semester. Respondents who did not meet the eligibility criteria did not proceed with the survey.

After completion, questionnaires were collected on site. A total of 1200 questionnaires were distributed, of which 1174 were returned. The returned questionnaires were subsequently screened, and responses were excluded if they failed to meet the eligibility criteria, showed patterned responding, contained substantial missing data on key items, or exhibited logical inconsistencies. The final analytic sample consisted of 1027 valid questionnaires. The demographic characteristics of the sample are presented in Table 1.

### 3.2. Measurement of Variables

The four core variables examined in this study were measured using scales with established reliability and validity as documented in the literature. All scales employ a 5-point Likert scale, where “1” represents “strongly disagree” and “5” represents “strongly agree”; higher scores indicate a stronger manifestation of the variable. The selection of each scale was based on its applicability to the Chinese college student population, the completeness of its theoretical framework, and its demonstrated reliability and validity in previous research, as detailed below. For the measurement of group music activity participation, we adopted the scale used in previous research (Yang et al., 2025). This scale was developed based on the School Engagement Scale (Fredricks et al., 2004) and incorporated a three-dimensional framework for social activity participation among college students (J. Tang, 2017), namely, behavioral engagement, psychological engagement, and social interaction engagement, ultimately resulting in four items suitable for group music activity contexts. This scale focuses on the extent to which individuals actively participate in and engage with group music activities. In this study, typical items on the scale include “I actively participate in group music activities organized by the school” and “I practice and perform carefully in group music activities.”

Regarding the measurement of social connectedness, this study adopted the four-item scale used in previous research (Yang et al., 2025). The measure was adapted from prior work on social connection, including the UBC State Social Connection Scale (Lok & Dunn, 2023). The original UBC-SSCS contains 10 items and was designed to assess state social connection within a specified time frame. In contrast, the four-item version used by Yang et al. and adopted in the present study does not specify a momentary reference period or restrict responses to a particular music-group context. Accordingly, in the present study, social connectedness refers to participants’ relatively general perceptions of interpersonal belonging, acceptance, enjoyment, and being valued in their relationships with others. The four items were: “I feel that I am part of a community of friends,” “I find connections with friends enjoyable and fulfilling,” “I feel accepted and understood when I interact with others,” and “My friends make me feel valued and respected.” All items were rated on a five-point Likert scale, with higher scores indicating stronger social connectedness.

Regarding the measurement of collective effervescence, this study employed the Collective Effervescence Scale developed by Gabriel et al. and revised by Chinese scholars Xue and Yin (Gabriel et al., 2020; Xue & Yin, 2024). Consisting of eight items, the scale measures the degree of collective emotional fusion perceived by individuals when participating in community music activities. Before presenting the scale, respondents were instructed to recall their experiences in group music activities over the past semester and to rate each item based on how they generally felt during those activities. This instruction ensured that all respondents referred to a common reference period, namely the past semester, thereby making their reports comparable across individuals. Accordingly, in the present study, Collective Effervescence was measured as students’ retrospective perceptions of collective emotional experience in group music activity contexts during the past semester, rather than as a direct real-time measure of emotional synchronization as it unfolded during the activity itself. Typical items include “I feel that most participants share the same emotional experience” and “I feel that this activity seems somewhat special.”

Measuring Academic Burnout: Academic burnout was measured using the College Student Academic Burnout Scale (Lian et al., 2005). This scale was constructed based on Maslach and Leiter’s three-factor model of occupational burnout and has been widely used among Chinese college students, with satisfactory reliability and validity (Maslach & Leiter, 2000). The scale consists of 20 items rated on a 5-point Likert scale and comprises three dimensions: Emotional Dejection (8 items; e.g., “After a full day of studying, I feel completely exhausted”), Improper Learning Behavior (6 items; e.g., “I rarely plan or organize my study time”), and Reduced Personal Accomplishment (6 items; e.g., “I can handle university courses well,” reverse-scored). Emotional Dejection comprises Items 2, 4, 5, 7, 9, 12, 17, and 20; Improper Learning Behavior comprises Items 1, 8, 10, 14, 16, and 19; and Reduced Personal Accomplishment comprises Items 3, 6, 11, 13, 15, and 18. Items 1, 3, 6, 8, 11, 13, 15, and 18 are reverse-worded and were reverse-scored prior to analysis, with responses recoded as 1 = 5, 2 = 4, 3 = 3, 4 = 2, and 5 = 1, so that higher scores on all items consistently indicate higher levels of academic burnout; the remaining 12 items were scored directly. This reverse-scoring procedure is identical to that specified in the original scale. All scales have been validated among a sample of Chinese college students and demonstrate sound reliability and validity.

To further ensure the applicability and content validity of the measurement tools in the context of this study, the research team invited two experts—both professors holding PhD in psychology—to review each scale item individually. The experts provided suggestions for revisions regarding the clarity of item wording, consistency with the conceptual content, and cultural appropriateness within the study population. Based on these recommendations, the research team made minor adjustments to some items to better align with the context of group music activities. Prior to the formal administration, a small-scale pilot study (N = 58) was conducted to preliminarily assess the internal consistency reliability of the scales. The results showed that the Cronbach’s α coefficients for each scale ranged from 0.738 to 0.916, indicating excellent reliability levels within the study sample. The results of the pilot study were not included in the formal analysis.

### 3.3. Ethics and Informed Consent

This study adheres to the principles of the Declaration of Helsinki and has been approved by the Ethics Committee of Southeast University (Approval No.: IRBSU20250129). All participants signed a written informed consent form.

### 3.4. Abbreviations

For ease of presentation, the following abbreviations are used for the main variables and dimensions in this study: GMAP stands for Group Music Activity Participation, SC stands for Social Connectedness, CE stands for Collective Effervescence, and AB stands for Academic Burnout, which comprises three second-order dimensions: ED (Emotional Dejection), ILB (Improper Learning Behavior), and RPA (Reduced Personal Accomplishment).

### 3.5. Research Steps

All analyses were conducted using SPSS 26.0 and AMOS 24.0, with fsQCA 3.0 used for the configurational analyses. The measurement and structural models were estimated using maximum likelihood (ML). Given the five-category response format, items were treated as approximately continuous for ML estimation. This treatment was supported by the observed distributional diagnostics (item-level absolute skewness ≤ 0.64 and absolute kurtosis ≤ 1.21) and the large sample size (N = 1027). No item-level responses were missing in the final analytic dataset; therefore, no imputation or missing-data deletion procedures were required. Model identification followed the marker-variable method, with one indicator loading per latent factor fixed to 1.0. No post hoc modifications were made to the measurement or structural models. For the mediation analysis, bias-corrected percentile bootstrap confidence intervals were computed using 5000 resamples. For the primary fsQCA, composite scores were calculated as unweighted item averages and calibrated using the direct method with the 95th, 50th, and 5th percentile anchors. The truth table was constructed using a consistency threshold of 0.80, a PRI threshold of 0.75, and a frequency threshold of 16 cases.

## 4. Results

### 4.1. Descriptive Statistics and Confirmatory Factor Analysis (CFA)

Descriptive statistics and distributional diagnostics for the study measures are reported in Table 2. The mean scores ranged from 3.46 for RPA to 3.69 for SC on the 1 to 5 response scale, and the distributions showed no serious departures from normality (skewness from −0.39 to 0.05, kurtosis from −1.00 to −0.09). At the item level, the absolute skewness and kurtosis of all 36 items did not exceed 0.64 and 1.21, respectively, supporting the use of maximum likelihood estimation with the five-category items treated as continuous. Internal consistency in the present sample was acceptable to good (Cronbach’s α from 0.877 to 0.907). Participants ranged in age from 18 to 26 years (M = 20.33, SD = 1.44).

Confirmatory factor analysis (CFA) is used to examine the convergent and discriminant validity of latent variables and their corresponding measurement items in a research model, ensuring the reliability and validity of the measurement instrument and laying an empirical foundation for subsequent hypothesis testing. The convergent validity of each latent variable is assessed using standard loadings, average variance extracted (AVE), and composite reliability (CR). Standard loadings reflect the extent to which measurement items load onto the latent variable, AVE measures the latent variable’s ability to explain variance in the measurement items, and CR evaluates the internal consistency of the measurement items. The test results show that the standard loadings of the measurement items for each latent variable all meet the critical threshold of 0.7 or higher. The standard loadings of the four measurement items for the GMAP latent variable are 0.830, 0.789, 0.806, and 0.805, respectively, with an AVE of 0.652 exceeding the 0.5 threshold and a CR of 0.882 exceeding the 0.7 standard, indicating that the measurement scale has good convergent validity and internal consistency. The standard loadings for the four items of the SC latent variable range from 0.790 to 0.808; the AVE of 0.642 and the CR of 0.878 both meet the standards, indicating that the items exhibit strong convergent validity and can reliably reflect the core dimensions of the latent variable. The standard loadings of the eight measurement items for the CE latent variable range from 0.716 to 0.764. The AVE value of 0.548 is slightly above the 0.5 threshold, and the CR value of 0.907 is significantly higher than 0.7, demonstrating extremely high internal consistency. The standard loadings for the eight items of the EX latent variable range from 0.711 to 0.745. The AVE value of 0.529 and the CR value of 0.900 both meet the requirements, indicating that the items effectively capture its core meaning. For the RE latent variable, the standard loadings of the six measurement items ranged from 0.716 to 0.812. The AVE of 0.561 and the CR of 0.885 met the standards, indicating reliable internal consistency of the scale. For the RPA latent variable, the standard loadings of the six measurement items range from 0.706 to 0.758. The AVE value of 0.543 and the CR value of 0.877 meet the requirements, effectively reflecting the characteristics of the latent variable (Table 3).

### 4.2. Common Method Bias

To assess the severity of common method bias, this study employed two methods. First, a Harman single-factor test was conducted, performing unrotated principal component analysis on all measurement items. The results showed that the first unrotated factor explained 28.85% of the variance, which is below the critical threshold of 40%, indicating that there is no severe common method bias. Second, confirmatory factor analysis (CFA) was used for comparison. This study compared the unifactor model with the measurement model. As shown in the model fit index test results in Table 4, there was a significant difference in the fit between the unifactor model and the measurement model. The chi-square value for the unifactor model was 10,972.963 with 594 degrees of freedom, corresponding to a chi-square-to-degrees-of-freedom ratio of 18.47, far exceeding the recommended threshold of 3, indicating that the model fits extremely poorly. In contrast, the chi-square value for the measurement model dropped to 984.890, with a chi-square-to-degrees-of-freedom ratio of 1.701, falling within the ideal range of 1 to 3, suggesting that the measurement model has good overall fit and strong compatibility with the data. The Root Mean Square Error of Approximation (RMSEA) for the measurement model is 0.026 (90% CI [0.023, 0.029]), which is below the standard of 0.05, suggesting excellent approximate fit. In contrast, the RMSEA for the single-factor model is 0.130, far exceeding the critical value of 0.1, indicating severe fit bias in that model. Regarding the Goodness-of-Fit Index (GFI), the measurement model’s GFI value is 0.946, approaching the perfect fit standard of 1, reflecting that the model can explain the data’s variance well. In contrast, the single-factor model’s GFI value is only 0.461, indicating extremely low explanatory power for the data. The results for the Standardized Root Mean Square Residual (SRMR), Normed Fit Index (NFI), Tucker–Lewis Index (TLI), and Comparative Fit Index (CFI) further corroborate this difference. The SRMR for the measurement model is 0.027, with NFI, TLI, and CFI values of 0.950, 0.977, and 0.979, respectively, all exceeding the acceptable threshold of 0.9 and approaching the excellent standard of 0.95. In contrast, the corresponding indices for the single-factor model are all far below the acceptable threshold. Considering both methods together, this study does not exhibit serious common method bias issues.

### 4.3. Discriminant Validity

Discriminant validity was assessed by comparing the square root of the average variance extracted (AVE) of each latent variable with the absolute values of its correlations with the other latent variables. The criterion was that the square root of the AVE of each latent variable must be greater than its correlations with all other latent variables, in order to avoid dimensional overlap. As shown in Table 5, the largest absolute correlation of each latent variable was smaller than its square root of AVE (GMAP: 0.462 < 0.808, SC: 0.463 < 0.802, CE: 0.463 < 0.740, ED: 0.412 < 0.727, ILB: 0.491 < 0.749, and RPA: 0.491 < 0.737). All latent variables met the criterion, and the discriminant validity test was passed. In summary, all indicators of this confirmatory factor analysis meet the conventional standards, and the measurement scales demonstrate good convergent validity, internal consistency reliability, and discriminant validity, providing an adequate empirical basis for the subsequent hypothesis testing and structural modeling.

### 4.4. Correlation

The correlation results are presented in Table 6. GMAP showed a significant positive correlation with both SC and CE, with correlation coefficients of 0.436 and 0.435, respectively, both reaching the 0.01 significance level. This indicates that higher GMAP levels were accompanied by higher levels of SC and CE, suggesting a relatively strong positive association among the three variables. SC and CE also showed a significant positive correlation, with a correlation coefficient of 0.431 that is significant at the 0.01 level, suggesting that the two variables were positively associated with each other. ED shows a significant negative correlation with GMAP, SC, and CE, with correlation coefficients of −0.343, −0.375, and −0.340, respectively, and all are significant at the 0.01 level. This indicates that higher levels of ED were associated with lower levels of GMAP, SC, and CE. ILB also showed significant negative correlations with GMAP, SC, and CE, with correlation coefficients of −0.360, −0.377, and −0.379, and these were also significant at the 0.01 level, indicating negative associations between ILB and these variables. Meanwhile, ILB showed a significant positive correlation with ED, with a correlation coefficient of 0.352 that was significant at the 0.01 level, indicating a positive association between ILB and ED. RPA showed significant negative correlations with GMAP, SC, and CE, with correlation coefficients of −0.364, −0.287, and −0.335, respectively, all reaching the 0.01 significance level. This indicates that higher RPA levels were associated with lower levels of GMAP, SC, and CE. At the same time, RPA showed a significant positive correlation with ED, with a correlation coefficient of 0.181 and significance at the 0.01 level. It also showed a significant positive correlation with ILB, with a correlation coefficient of 0.433 and significance at the 0.01 level. This indicates that the positive association between RPA and ILB is stronger, while the positive association with ED is relatively weaker. It should also be noted that Table 6 reports Pearson correlations among the observed composite scores, whereas Table 5 reports the correlations among the latent variables estimated in the confirmatory factor analysis. The latent correlations correct for measurement error and are therefore slightly larger in magnitude than the corresponding observed correlations (for example, the GMAP-SC correlation is 0.462 in Table 5 and 0.436 in Table 6). Both sets of estimates show the same pattern of signs and relative magnitudes.

### 4.5. Results of the Structural Equation Model

The unadjusted structural model showed good fit to the data, χ^2^(585) = 1045.77, χ^2^/df = 1.787, CFI = 0.976, TLI = 0.974, RMSEA = 0.028, 90% CI [0.025, 0.030], and SRMR = 0.039. The model explained 21.3% of the variance in SC, 27.5% in CE, and 60.4% in AB. The covariate-adjusted model also showed comparable fit (Table 7).

As shown in Table 8, the results of the structural equation model indicate that all focal paths among the latent variables were statistically significant. Given the cross-sectional and self-report nature of the data, these path coefficients should be interpreted as associations consistent with the proposed theoretical model, rather than as evidence of causal relationships or temporal ordering. Specifically, the path from GMAP to SC was significant, with a standardized coefficient of 0.462. This suggests that higher levels of Group Music Activity Participation were associated with higher levels of Social Connectedness. The path from GMAP to CE was also significant, with a standardized coefficient of 0.279, indicating that higher levels of Group Music Activity Participation were associated with higher levels of retrospectively perceived Collective Effervescence in group music activity contexts. In addition, the path from SC to CE was significant, with a standardized coefficient of 0.334. This result suggests that students reporting stronger Social Connectedness also tended to report stronger retrospective perceptions of Collective Effervescence.

Regarding the associations with Academic Burnout, the path from GMAP to AB was significant and negative, with a standardized coefficient of −0.310. This indicates that higher levels of Group Music Activity Participation were associated with lower levels of Academic Burnout. The path from SC to AB was also significant and negative, with a standardized coefficient of −0.299, suggesting that stronger Social Connectedness was linked to lower Academic Burnout. The path from CE to AB was significant and negative as well, with a standardized coefficient of −0.365. This suggests that stronger retrospectively perceived Collective Effervescence was associated with lower Academic Burnout, and its standardized association with AB was slightly stronger than those of GMAP and SC in the present model.

**Figure 2:**
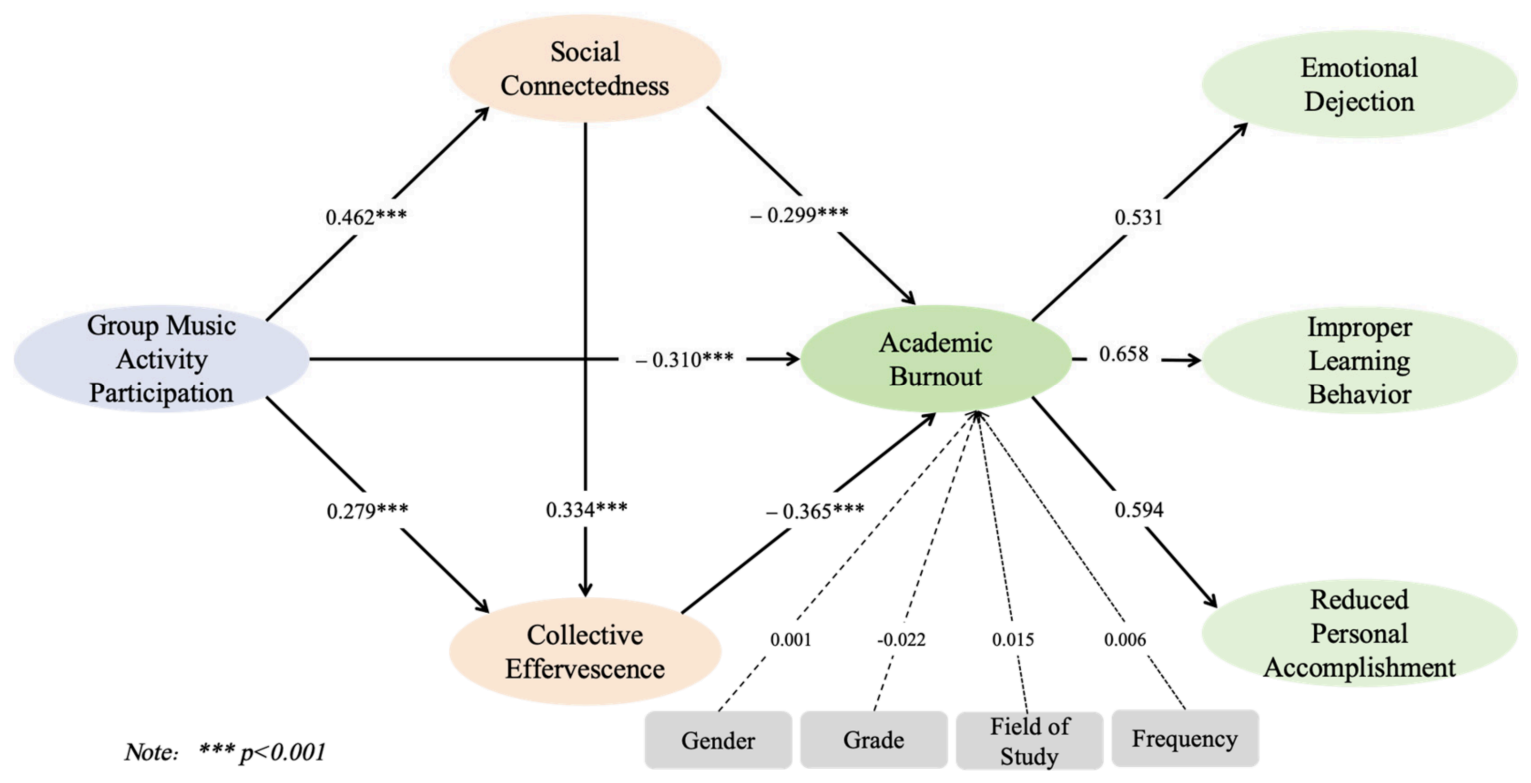
Diagram of the revised structural equation model.

For the measurement paths of Academic Burnout, the unstandardized coefficient from AB to ED was fixed at 1.000 as the reference loading, with a standardized coefficient of 0.531. The standardized coefficients from AB to ILB and RPA were 0.658 and 0.594, respectively, and both reached statistical significance. These results indicate that Emotional Dejection, Improper Learning Behavior, and Reduced Personal Accomplishment all served as meaningful observed indicators of the latent construct of Academic Burnout, with Improper Learning Behavior showing the highest standardized loading among the three dimensions. In the measurement model for GMAP, the unstandardized coefficient from GMAP to GMAP1 was fixed at 1.000 as the reference loading, with a standardized coefficient of 0.830. The standardized coefficients for GMAP2, GMAP3, and GMAP4 were 0.789, 0.806, and 0.805, respectively. The standardized loadings of the GMAP indicators ranged from 0.789 to 0.830 and all reached statistical significance, suggesting that the observed indicators adequately represented the latent construct of Group Music Activity Participation. The revised structural equation model diagram is shown in Figure 2.

The model was re-estimated with Gender, Grade, Field of Study, and Frequency of participation in group music activities entered as covariates predicting Academic Burnout. The covariate-adjusted model also showed acceptable fit (Table 7): chi-square = 1204.16 with 725 degrees of freedom, chi-square-to-degrees-of-freedom ratio = 1.66, CFI = 0.975, TLI = 0.973, RMSEA = 0.025 (90% CI [0.023, 0.028]), and SRMR = 0.037. None of the four covariates was significantly associated with AB (Gender: b = 0.001, *p* = 0.977, Grade: b = −0.016, *p* = 0.480, Field of Study: b = 0.010, *p* = 0.621, Frequency: b = 0.009, *p* = 0.856). The focal paths were essentially unchanged after the inclusion of these covariates (for example, GMAP → AB: standardized coefficient = −0.314 versus −0.310 in the unadjusted model), indicating that the core results did not depend on the inclusion of the control variables.

### 4.6. Mediation Analysis

This study employed the bias-corrected percentile bootstrap method, conducting 5000 bootstrap repetitions to calculate the 95% confidence intervals for each indirect path estimate. All estimates reported in Table 9 are standardized. If the confidence interval does not include 0, the indirect path is considered statistically significant. Because the bootstrap procedure displays *p*-values at a resolution of 0.001, values at or below this floor are reported as *p* < 0.001 rather than as exact values, and the bias-corrected confidence intervals are the primary basis for inference. In line with the cross-sectional design of the study, the mediation results are interpreted as indirect associations that are consistent with the proposed mediation model, rather than as evidence of causal mediation. The results of the mediation analysis are shown in Table 9.

Specifically, the chain indirect path of GMAP → SC → CE → AB was statistically significant, with an estimated value of −0.056 and a 95% confidence interval of [−0.084, −0.036], which does not include 0. This result suggests that higher levels of Group Music Activity Participation were indirectly associated with lower levels of Academic Burnout through higher Social Connectedness and stronger retrospectively perceived Collective Effervescence in sequence. This finding is consistent with the proposed chain mediation model, but should be interpreted as a statistically significant sequential indirect association involving Social Connectedness and Collective Effervescence rather than as evidence of a temporal psychosocial process.

The indirect path of GMAP → SC → AB was also statistically significant, with an estimated value of −0.138 and a 95% confidence interval of [−0.198, −0.088]. Since this interval does not include 0, a statistically significant indirect association through Social Connectedness was observed between Group Music Activity Participation and Academic Burnout. More specifically, higher GMAP was associated with higher SC, and higher SC was in turn associated with lower AB. This result supports the view that social connectedness may be an important relational resource involved in the association between group music activity participation and academic burnout.

The indirect path of GMAP → CE → AB was also statistically significant, with an estimated value of −0.102 and a 95% confidence interval of [−0.148, −0.068], which does not include 0. Similarly, a statistically significant indirect association through Collective Effervescence was observed between Group Music Activity Participation and Academic Burnout. Higher levels of GMAP were associated with stronger retrospective perceptions of CE, and stronger CE was associated with lower AB. This result is consistent with the theoretical expectation that shared emotional experience in group music activity contexts may be related to lower levels of academic burnout, while it should not be interpreted as direct evidence that such emotional experience causally reduces burnout.

The direct path from GMAP to AB was statistically significant, with an estimated value of −0.310 and a 95% confidence interval of [−0.408, −0.211], which does not include 0. This indicates that Group Music Activity Participation remained directly associated with lower Academic Burnout after the inclusion of Social Connectedness and Collective Effervescence in the model. The total association between GMAP and AB was also statistically significant, with an estimated value of −0.606 and a 95% confidence interval of [−0.707, −0.512], which does not include 0. The total estimate was composed of the direct path and the three indirect paths, namely −0.310 + (−0.138) + (−0.102) + (−0.056) = −0.606. Overall, the direct path analysis and mediation analysis provided mutually consistent results. Group Music Activity Participation was associated with Academic Burnout both directly and indirectly through Social Connectedness and Collective Effervescence. The significant indirect paths suggest that SC and CE may be important psychosocial variables involved in this association. Rather than demonstrating a causal mechanism, these findings should be understood as evidence that is statistically consistent with the proposed theoretical model, and that provides a basis for future longitudinal, experimental, or experience-sampling research to further examine temporal ordering and causal mediation.

### 4.7. Fuzzy Set Qualitative Comparative Analysis (fsQCA)

Before conducting fsQCA, the latent variables were converted into observable composite scores. Each composite was computed as the unweighted average of its item scores, which places all composites on the same 1 to 5 metric. Because the three dimensions of Academic Burnout contain different numbers of items (8, 6, and 6), the overall burnout composite, that is, the average of all 20 items, weights each dimension in proportion to its item count. Using item sums instead of averages yields identical calibrated membership scores, because both the scores and the percentile anchors are rescaled by the same constant. Calibration used the direct method following Ragin (2009) and Fiss (2011). The 95th, 50th, and 5th percentiles of the sample distribution served as the anchors for full membership, the crossover point, and full non-membership, respectively, converting the scores into fuzzy membership values in the range of 0 to 1. These percentile anchors indicate relative set membership within the present sample rather than externally validated substantive or clinical thresholds. Following common practice, a constant of 0.001 was added to all membership values so that no case would receive a membership score of exactly 0.5. All values remained within the interval [0, 1] after this adjustment (maximum = 0.956, minimum = 0.002). The negated sets, denoted by a tilde, were computed as 1 minus the corresponding membership score, so that ~AB, for example, represents low academic burnout. The calibration anchors are shown in Table 10.

The necessity analysis is evaluated based on a consistency coefficient of ≥0.90, supplemented by a coverage coefficient of >0.6. (Mattke et al., 2021). As shown in Table 11, the consistency of all conditions and their negations falls short of 0.90. Although GMAP, SC, and CE exhibit high coverage, they do not constitute necessary conditions for ~AB, indicating that ~AB is more likely to result from the combined effects of multiple conditions (Table 12).

When constructing the truth table, the consistency threshold was set to 0.80 and the PRI threshold to 0.75. Following the recommendations of Schneider and Wagemann (2012), the case frequency threshold was set to 1.5% of the total sample size (1027), meaning that configurations with a frequency below 16 cases would be excluded. No configuration was excluded by this criterion, because all eight configurations contained at least 65 cases. The complete truth table is reported in Table 13. Only the configuration combining high GMAP, high SC, and high CE passed both thresholds (consistency = 0.896, PRI = 0.799) and was coded as 1. The remaining configurations were coded as 0, either because their consistency fell below 0.80 (the all-absent configuration) or because they failed the PRI criterion despite adequate consistency (all other configurations). Because all eight configurations are empirically populated, the truth table contains no logical remainders, and the counterfactual analysis used to derive the intermediate solution therefore did not influence the result. Directional expectations for the intermediate solution specified the presence of all three conditions, consistent with the theoretical framework. The Quine-McCluskey minimization yielded a single configuration, GMAP*SC*CE. The configuration consistency was 0.896, with raw and unique coverage both equal to 0.647. The overall solution consistency was 0.896, and the overall coverage was 0.647, indicating that this configuration accounted for approximately 64.7% of the low-burnout cases.

Sensitivity analyses are reported in Table 14. The solution was re-estimated under alternative calibration anchors (90th/50th/10th and 85th/50th/15th percentiles), alternative consistency thresholds (0.75 and 0.85), an alternative PRI threshold (0.70), and alternative frequency thresholds (1, 5, 10, and 30 cases). In every setting, the same single configuration (GMAP*SC*CE) was retained with comparable consistency and coverage. The configuration ceased to be retained only when a threshold exceeded the configuration’s own values; that is, at a consistency threshold of 0.90 or a PRI threshold of 0.80 against the configuration values of 0.896 and 0.799, which is a boundary property of the solution rather than evidence of instability.

Following the logic of causal asymmetry, the analysis was repeated with high academic burnout (AB) as the outcome. A single sufficient configuration was identified, ~GMAP*~SC*~CE (consistency = 0.916, coverage = 0.522), indicating that high burnout was consistently associated with the simultaneous absence of high participation in group music activities, high social connectedness, and high collective effervescence. This mirrored pattern complements the low-burnout solution and is consistent with the joint relevance of the three conditions for students’ burnout status.

## 5. Discussion

### 5.1. Group Music Activity Participation and Academic Burnout

Higher levels of group music activity participation were significantly associated with lower academic burnout, which is broadly consistent with previous research linking collective music participation to favorable psychosocial and academic outcomes. Jiang (2025) reported that group music activity participation was associated with stronger social-emotional competence and lower academic burnout among college students, while other studies have linked group musical participation to better social adjustment and interpersonal connectedness (Qiao, 2026) and choral participation to greater emotional, psychological, and social well-being (Acquah, 2016). Participation alone, however, may not be enough to explain lower burnout. Octaviani et al. (2023) found that students involved in organizational activities could still experience academic burnout, suggesting that the nature of the participation may matter. Group music activities involve shared presence, rhythmic coordination, mutual responsiveness, and emotional expression, which may make relational and emotional processes especially salient. Repeated coordination and responsiveness to others may strengthen interpersonal connection and reduce feelings of isolation, which could be relevant for students coping with prolonged academic pressure. The present findings therefore extend previous work on the psychosocial benefits of group music participation to academic burnout among non-music-major university students, although the cross-sectional self-report design does not support a causal interpretation of this association.

### 5.2. Mediating Roles of Social Connectedness and Collective Effervescence

The findings support the proposed indirect associations of social connectedness and collective effervescence in the relationship between group music activity participation and academic burnout, reflecting two distinct but complementary psychosocial experiences of collective participation. Social connectedness has been identified as an important resource in stressful contexts. Higher levels of social connectedness have been associated with lower examination stress (Mickley Steinmetz et al., 2024), lower loneliness and perceived stress (L. Tang et al., 2025), and reduced negative psychological consequences of adverse social experiences (Sun et al., 2021). Weakened social connectedness has also been linked to greater burnout under stressful conditions (Yıldırım et al., 2021). Group music activities involve repeated interaction, coordination, and mutual responsiveness, which may foster feelings of belonging, acceptance, and relational integration. Social connectedness may therefore represent a relational resource associated with both participation in group music activities and lower academic burnout.

Collective effervescence reflects the emotional dimension of shared musical experiences, particularly the heightened emotional energy and sense of unity that can emerge through collective participation. Previous research has linked collective effervescence in live and group contexts to enhanced well-being, sustained positive emotional experience, social integration, and group identity (Wlodarczyk et al., 2020; Naidu et al., 2024; Koefler et al., 2026). Group music activities may be especially conducive to such experiences because they involve shared presence, rhythmic coordination, and emotional resonance. These experiences may be associated with greater subjective emotional resources and lower perceived exhaustion, providing a possible explanation for the observed association with academic burnout. However, the findings indicate statistical indirect associations rather than established psychological mechanisms, and the cross-sectional design does not allow causal direction to be determined.

### 5.3. Serial Indirect Association of Social Connectedness and Collective Effervescence

The serial indirect association through social connectedness and collective effervescence is a distinctive feature of the proposed model. Previous research has linked group music participation with social connection, shared emotional experience, and psychological well-being, but these processes have more often been examined separately. The present findings bring the relational and emotional dimensions of group participation into the same framework. Social connectedness may capture how participation becomes a sense of relational integration, as repeated coordination and mutual responsiveness in group music activities can be associated with stronger feelings of belonging and psychological connection with others. Collective effervescence reflects a shared emotional experience characterized by emotional synchronization and group unity. Conceptually, this distinction can be understood as a shift from “I belong to this group” to “we are experiencing this together.” This interpretation is consistent with research showing that shared attention, co-presence, and interaction are associated with emotional synchronization and group attachment, while live-music experiences have also been linked to social connectedness and psychological and social well-being (Páez et al., 2015; Packer & Ballantyne, 2011; Zabala et al., 2023). The results suggest that these relational and emotional experiences may operate in combination when considering the association between group music participation and academic burnout. The proposed sequence should not, however, be treated as a confirmed temporal process. Cross-sectional data cannot establish that social connectedness precedes collective effervescence, and the reverse relationship is also plausible, with shared emotional experiences potentially strengthening feelings of connection. The ordering tested here is one theoretically grounded model that requires further examination with longitudinal or experimental data.

### 5.4. Configurational Findings

The fsQCA results complement the SEM findings by showing that high group music activity participation, high social connectedness, and high collective effervescence formed a sufficient configuration associated with low academic burnout, while no single condition was necessary on its own. This pattern suggests that low academic burnout may be related to the joint presence of behavioral, relational, and emotional conditions rather than to any one factor in isolation. Group music activity participation reflects engagement in a collective setting, social connectedness captures the relational experience of interaction and belonging, and collective effervescence reflects the shared emotional experience that may emerge through coordinated participation. Their joint presence suggests that the relevance of group music activities may depend not only on participation itself but also on the quality of the relational and emotional experiences surrounding it. At the same time, the identified configuration may partly reflect the characteristics of the present sample and the analytical choices involved in calibration, consistency thresholds, and case-frequency criteria. Because the sample consisted of non-music-major college students who had participated in group music activities during the previous semester, the range of conditions may also have been restricted. The configuration should therefore be interpreted as one sufficient pattern observed under the present sample and methodological conditions, rather than as evidence that other configurations cannot be associated with low academic burnout. SEM and fsQCA address different aspects of the same relationships: SEM estimates the associations specified in the proposed model, including the indirect associations involving social connectedness and collective effervescence, whereas fsQCA identifies combinations of conditions associated with low academic burnout. Using both approaches allows these relationships to be examined from variable-centered and configurational perspectives.

### 5.5. Theoretical Contributions and Practical Implications

The present study contributes to the literature by extending research on group music participation to academic burnout among non-music-major university students. Previous studies have largely focused on outcomes such as social adjustment, interpersonal connection, and psychological well-being, while academic burnout research has more often emphasized academic stress, individual psychological resources, and general social support. Examining these areas together places group music participation within the broader psychosocial context of students’ academic adjustment. The study also considers social connectedness and collective effervescence within the same framework, bringing together relational processes such as belonging and social integration with emotional processes such as shared activation and synchronization. The proposed serial indirect pattern offers a possible account of how group music participation may be associated with relational integration, shared emotional experience, and academic burnout. Methodologically, combining SEM and fsQCA provides complementary perspectives: SEM examines associations within the hypothesized model, whereas fsQCA identifies configurations of conditions associated with low academic burnout. This makes it possible to consider both variable-level relationships and the joint occurrence of group music participation, social connectedness, and collective effervescence.

The findings also have practical implications for universities considering group music activities as part of student well-being initiatives. The relational and shared emotional qualities of participation may be as relevant as participation itself. Activities that encourage sustained interaction, coordination, mutual responsiveness, and a sense of belonging may provide richer psychosocial experiences, while opportunities for meaningful shared emotional experiences may further strengthen students’ engagement in collective music activities. These implications remain provisional, however, because the cross-sectional design does not establish that group music activities or the proposed psychosocial processes have causal or intervention effects on academic burnout.

### 5.6. Limitations and Future Directions

Several limitations should be considered when interpreting the findings. Most importantly, the cross-sectional self-report design does not permit conclusions about causal or temporal ordering among group music activity participation, social connectedness, collective effervescence, and academic burnout. Reverse or reciprocal relationships are also possible; for example, students with lower burnout or stronger social connectedness may be more inclined to participate in group music activities. Longitudinal or experimental designs would be needed to examine whether changes in participation precede changes in these psychosocial experiences or in academic burnout.

The sampling strategy also places important limits on interpretation. Participants had all taken part in at least one group music activity during the previous semester and were recruited largely through settings related to campus music activities. The findings therefore reflect variation among students who were already participating rather than a comparison between participants and non-participants, and should not be interpreted as evidence of the effect of participation itself. In addition, some participants may have belonged to the same universities, choirs, clubs, ensembles, or campus events. Because group-level identifiers were not retained after anonymization, possible clustering could not be assessed, which may have led to underestimated statistical uncertainty if meaningful within-group dependence was present. Future studies would benefit from including comparable non-participants and retaining de-identified organizational or activity-group information so that cluster-robust or multilevel analyses can be applied where necessary.

The present study also captured participation only in a relatively broad way. Although participation frequency was included as a control variable in the SEM analysis, differences between occasional and sustained participation were not examined directly. Regular participation may provide more opportunities for repeated coordination, group cohesion, and shared emotional experience than sporadic attendance. Similarly, the specific type of music activity was used mainly to describe the sample, so the study could not determine whether choir, orchestra, band rehearsal, music clubs, or group performances differ in their associations with social connectedness, collective effervescence, or academic burnout. Future work could examine participation frequency, duration, consistency, group size, coordination demands, and interaction intensity more systematically.

Measurement should also be interpreted with some caution. All major variables were assessed through self-report and may therefore be influenced by recall or social desirability. Although collective effervescence was framed specifically in relation to group music activities, it was measured retrospectively using relatively general items rather than through direct assessment of emotional synchronization during participation. Immediate post-activity reports, experience sampling, behavioral observation, or interpersonal and physiological measures may provide a more direct account of collective emotional experience. The study also did not compare group music participation with other collective activities such as sports, dance, drama, volunteering, or student clubs, so it remains unclear whether the observed associations are specific to music or reflect broader features of collective participation. Finally, the sample consisted of Chinese non-music-major university students, and further research across different educational and cultural contexts is needed to assess the extent to which these patterns generalize.

## 6. Conclusions

This study examined the associations among group music activity participation, social connectedness, collective effervescence, and academic burnout among non-music-major Chinese college students with recent experience in group music activities. Higher participation was associated with lower academic burnout, with social connectedness and collective effervescence showing significant indirect associations consistent with the proposed model. The serial indirect association through social connectedness and collective effervescence was also supported, although the cross-sectional design does not allow temporal or causal ordering to be established. The fsQCA analysis showed that no single condition was necessary for low academic burnout, while high group music activity participation, high social connectedness, and high collective effervescence jointly formed a sufficient configuration under the present sample and analytical conditions. This pattern represents one possible configuration rather than the only pathway to low academic burnout. The findings indicate that participation in group music activities may be related to academic burnout not only through participation itself, but also through the relational and shared emotional experiences associated with it. Combining SEM and fsQCA provides two complementary ways of examining these relationships, while longitudinal and experimental research is still needed to clarify their temporal and causal nature.

## Figures and Tables

**Table 1:** Demographic information of the sample population.

Variable	Option	Frequency (N)	Percentage (%)
Gender	Male	492	47.9
	Female	535	52.1
Grade	Freshman	231	22.5
	Sophomore	286	27.9
	Junior	298	29.0
	Senior	189	18.4
	Graduate student and above	23	2.2
Field of Study	Science and Engineering	371	36.1
	Humanities and Social Sciences	403	39.2
	Business and Management	186	18.1
	Arts (Apart from music majors)	45	4.4
	Other	22	2.2
Frequency of participation in group music activities	1–2 times a month	412	40.1
	3–4 times a month	328	31.9
	3–5 times per semester	203	19.8
	1–2 times per semester	84	8.2
Types of group music activities (Multiple choice)	Choir or band rehearsals	489	47.6
	Campus singing competitions	376	36.6
	Music club activities	402	39.1
	Group performances at orientation parties or cultural shows	451	43.9
	Other	128	12.5

**Table 2:** Descriptive statistics of the study measures.

Measure	M	SD	Min	Max	Skewness	Kurtosis
GMAP	3.593	1.114	1	5	−0.280	−0.988
SC	3.692	1.135	1	5	−0.392	−1.004
CE	3.600	1.050	1	5	−0.028	−0.998
ED	3.482	1.040	1	5	0.053	−0.815
RE	3.563	1.016	1	5	−0.232	−0.896
RPA	3.457	1.050	1	5	0.004	−0.800
AB	3.499	0.753	1	5	0.004	−0.092

**Table 3:** Results of Confirmatory Factor Analysis.

Variable	Measurement Item	Standard Load Coefficient	AVE	CR
GMAP	GMAP1	0.830	0.652	0.882
	GMAP2	0.789		
	GMAP3	0.806		
	GMAP4	0.805		
SC	SC1	0.805	0.642	0.878
	SC2	0.790		
	SC3	0.803		
	SC4	0.808		
CE	CE1	0.739	0.548	0.907
	CE2	0.740		
	CE3	0.741		
	CE4	0.764		
	CE5	0.739		
	CE6	0.716		
	CE7	0.763		
	CE8	0.720		
ED	ED1	0.711	0.529	0.900
	ED2	0.743		
	ED3	0.716		
	ED4	0.726		
	ED5	0.745		
	ED6	0.720		
	ED7	0.728		
	ED8	0.728		
ILB	ILB1	0.808	0.561	0.885
	ILB2	0.812		
	ILB3	0.718		
	ILB4	0.718		
	ILB5	0.716		
	ILB6	0.716		
RPA	RPA1	0.746	0.543	0.877
	RPA2	0.706		
	RPA3	0.758		
	RPA4	0.729		
	RPA5	0.739		
	RPA6	0.743		

**Table 4:** Results of the model fit index test.

Fit Index	Single-Factor Model	Measurement Model
χ^2^	10,972.963	984.890
df	594	579
χ^2^/df	18.47	1.701
RMSEA	0.130	0.026
GFI	0.461	0.946
SRMR	0.128	0.027
NFI	0.448	0.950
TLI	0.428	0.977
CFI	0.461	0.979

**Table 5:** Discriminant validity: Correlation coefficients between latent variables and the square root of the average variances.

	GMAP	SC	CE	ED	ILB	RPA
GMAP	**0.808**					
SC	0.462	**0.802**				
CE	0.433	0.463	**0.740**			
ED	−0.353	−0.412	−0.379	**0.727**		
ILB	−0.362	−0.381	−0.397	0.321	**0.749**	
RPA	−0.380	−0.317	−0.374	0.204	0.491	**0.737**

Note: The diagonal elements (in bold) represent the square roots of the Average Variance Extracted (AVE) for each latent variable. Off-diagonal elements are the correlation coefficients between latent variables. Discriminant validity is established when the square root of AVE for each construct is greater than its correlations with all other constructs.

**Table 6:** Results of Pearson Correlation Analysis.

	GMAP	SC	CE	ED	ILB	RPA
GMAP	1					
SC	0.436 **	1				
CE	0.435 **	0.431 **	1			
ED	−0.343 **	−0.375 **	−0.340 **	1		
ILB	−0.360 **	−0.377 **	−0.379 **	0.352 **	1	
RPA	−0.364 **	−0.287 **	−0.335 **	0.181 **	0.433 **	1

Note: ** *p* < 0.01.

**Table 7:** Structural equation model fit tests: unadjusted model and covariate-adjusted model.

Common Metrics	Criteria	Unadjusted Model	Adjusted Model (With Covariates)
χ^2^	-	1045.77	1204.16
df	-	585	725
χ^2^/df	<3	1.787	1.661
GFI	>0.9	0.942	0.941
RMSEA	<0.10	0.028	0.025
RMSEA 90% CI	-	[0.025, 0.030]	[0.023, 0.028]
SRMR	<0.08	0.039	0.037
CFI	>0.9	0.976	0.975
NFI	>0.9	0.947	0.940
RFI	>0.9	0.943	0.936
TLI	>0.9	0.974	0.973
AGFI	>0.9	0.934	0.933
IFI	>0.9	0.976	0.975

Note: The unadjusted model corresponds to the structural model without covariates. The adjusted model additionally includes Gender, Grade, Field of Study, and Frequency of participation in group music activities as covariates predicting Academic Burnout.

**Table 8:** Path coefficients of the structural equation model.

Path	Path	Path	Unstandardized Coefficient	S.E.	C.R.	*p*	Standardized Coefficient
GMAP	→	SC	0.457	0.035	13.070	***	0.462
GMAP	→	CE	0.252	0.033	7.527	***	0.279
SC	→	CE	0.305	0.035	8.846	***	0.334
GMAP	→	AB	−0.151	0.023	−6.448	***	−0.310
SC	→	AB	−0.147	0.024	−6.133	***	−0.299
CE	→	AB	−0.196	0.027	−7.327	***	−0.365
AB	→	ED	1.000				0.531
AB	→	ILB	1.249	0.114	10.992	***	0.658
AB	→	RPA	1.148	0.111	10.344	***	0.594
GMAP	→	GMAP1	1.000				0.830
GMAP	→	GMAP2	0.957	0.034	27.972	***	0.789
GMAP	→	GMAP3	0.980	0.034	28.732	***	0.806
GMAP	→	GMAP4	0.963	0.034	28.712	***	0.805
Gender	→	AB	0.001	0.048	0.029	0.977	0.001
Grade	→	AB	−0.016	0.022	−0.707	0.480	−0.022
Field of Study	→	AB	0.010	0.021	0.494	0.621	0.015
Frequency	→	AB	0.009	0.047	0.181	0.856	0.006

Note: *** *p* < 0.001. Control paths are from the covariate-adjusted model.

**Table 9:** Results of the mediation analysis (standardized estimates).

Parameter	Estimate	SE	Lower	Upper	*p*
GMAP → SC → CE → AB ChainInd	−0.056	0.012	−0.084	−0.036	<0.001
GMAP → SC → AB Ind	−0.138	0.027	−0.198	−0.088	<0.001
GMAP → CE → AB Ind	−0.102	0.02	−0.148	−0.068	<0.001
GMAP → AB Direct	−0.310	0.051	−0.408	−0.211	<0.001
GMAP → AB Total	−0.606	0.049	−0.707	−0.512	<0.001

Note: All estimates are standardized. The confidence intervals are bias-corrected percentile bootstrap 95% confidence intervals based on 5000 resamples. Bootstrap *p*-values are reported at a resolution of 0.001, and values below this floor are shown as *p* < 0.001.

**Table 10:** Data Calibration Results Table.

		GMAP	SC	CE	AB
Percentiles	5	1.750	1.750	2.000	2.350
	50	3.500	3.750	3.375	3.450
	95	5.000	5.000	5.000	4.950

**Table 11:** Analysis of Necessary Conditions (Outcome variable: ~AB).

	Consistency	Coverage
GMAP	0.811	0.756
~GMAP	0.505	0.565
SC	0.803	0.754
~SC	0.495	0.548
CE	0.820	0.772
~CE	0.508	0.561

**Table 12:** ~AB Configuration Diagram.

	Configuration 1
GMAP	●
SC	●
CE	●
Raw coverage	0.647
Unique coverage	0.647
Consistency (of configuration)	0.896
Overall solution consistency	0.896
Overall solution coverage	0.647

● indicates the presence of a condition. Raw coverage refers to the proportion of cases explained by each configuration; unique coverage refers to the proportion of cases explained exclusively by each configuration; consistency indicates the degree to which a configuration is a sufficient condition for the outcome. Raw and unique coverage are equal because the solution contains a single configuration.

**Table 13:** Truth table for the low-burnout outcome (~AB).

GMAP	SC	CE	OUT	n	Consistency	PRI
0	0	0	0	247	0.578	0.146
0	0	1	0	80	0.861	0.465
0	1	0	0	73	0.867	0.460
0	1	1	0	65	0.884	0.496
1	0	0	0	76	0.845	0.393
1	0	1	0	84	0.875	0.485
1	1	0	0	81	0.865	0.476
1	1	1	1	321	0.896	0.799

Note: n = number of cases in the configuration. OUT = outcome assignment following the joint consistency (≥0.80) and PRI (≥0.75) criteria. All eight configurations are empirically populated, and no configuration was excluded by the frequency threshold (n ≥ 16).

**Table 14:** Sensitivity analyses of the fsQCA solution.

Sensitivity Check	Setting	Solution	Consistency	Coverage
Calibration anchors	90th/50th/10th percentiles	GMAP*SC*CE	0.897	0.615
Calibration anchors	85th/50th/15th percentiles	GMAP*SC*CE	0.894	0.590
Consistency threshold	≥0.75	GMAP*SC*CE	0.896	0.647
Consistency threshold	≥0.85	GMAP*SC*CE	0.896	0.647
PRI threshold	≥0.70	GMAP*SC*CE	0.896	0.647
Frequency threshold	≥1, 5, 10, 30 cases	GMAP*SC*CE	0.896	0.647

Note: Baseline settings are the 95th/50th/5th percentile anchors, a consistency threshold of 0.80, a PRI threshold of 0.75, and a frequency threshold of 16 cases. The configuration GMAP*SC*CE was retained under all alternative settings shown.

## Data Availability

The data presented in this study are available on request from the corresponding authors. Due to the nature of this study, which is based on questionnaire surveys containing sensitive personal information of participants (e.g., demographic characteristics, health status, etc.), the raw data cannot be made publicly available for privacy protection reasons, in accordance with ethical requirements. De-identified summary data may be available upon reasonable request to the corresponding author, subject to approval by the institutional ethics review board.
